# Inferring the *Brassica rapa* Interactome Using Protein–Protein Interaction Data from *Arabidopsis thaliana*

**DOI:** 10.3389/fpls.2012.00297

**Published:** 2013-01-04

**Authors:** Jianhua Yang, Kim Osman, Mudassar Iqbal, Dov J. Stekel, Zewei Luo, Susan J. Armstrong, F. Chris H. Franklin

**Affiliations:** ^1^University of BirminghamBirmingham, UK; ^2^University of NottinghamNottingham, UK

**Keywords:** *Brassica rapa*, *Arabidopsis thaliana*, interactome, protein–protein interaction, domain–domain interaction, meiosis

## Abstract

Following successful completion of the *Brassica rapa* sequencing project, the next step is to investigate functions of individual genes/proteins. For *Arabidopsis thaliana*, large amounts of protein–protein interaction (PPI) data are available from the major PPI databases (DBs). It is known that Brassica crop species are closely related to *A. thaliana*. This provides an opportunity to infer the *B. rapa* interactome using PPI data available from *A. thaliana*. In this paper, we present an inferred *B. rapa* interactome that is based on the *A. thaliana* PPI data from two resources: (i) *A. thaliana* PPI data from three major DBs, BioGRID, IntAct, and TAIR. (ii) ortholog-based *A. thaliana* PPI predictions. Linking between *B. rapa* and *A. thaliana* was accomplished in three complementary ways: (i) ortholog predictions, (ii) identification of gene duplication based on synteny and collinearity, and (iii) BLAST sequence similarity search. A complementary approach was also applied, which used known/predicted domain–domain interaction data. Specifically, since the two species are closely related, we used PPI data from *A. thaliana* to predict interacting domains that might be conserved between the two species. The predicted interactome was investigated for the component that contains known *A. thaliana* meiotic proteins to demonstrate its usability.

## Introduction

For *Arabidopsis thaliana*, large amounts of protein–protein interaction (PPI) data are available from the major PPI databases (DBs; Galperin and Fernandez-Suarez, 2012), for example BioGRID (Stark et al., 2006) and IntAct (Aranda et al., 2010). The volume of these PPI data continues to increase with information from recently published articles (Arabidopsis Interactome Mapping Consortium, 2011). Assuming the same rate of interaction as in budding yeast, researchers estimate that the protein products of the *A. thaliana* genome participate in approximately 200,000 PPIs, a large proportion of which are yet to be validated (Lin et al., 2009). Therefore, efforts have been made to predict PPIs at the level of the entire *A. thaliana* genome, i.e., to produce a predicted interactome (Geisler-Lee et al., 2007; Cui et al., 2008; Morsy et al., 2008; Lee et al., 2010; Lin et al., 2010; Gu et al., 2011). Broadly speaking, two types of strategies can be applied. One approach is based on functional conservation between orthologous proteins, so called “interologs,” where *A. thaliana* protein orthologs in other species are first predicted and interacting orthologs reveal possible interactions in *A. thaliana*. An example of this type of work was reported by Geisler-Lee et al. (2007), where they surveyed PPI data in budding yeast (*Saccharomyces cerevisiae*), nematode worm (*Caenorhabditis elegans*), fruitfly (*Drosophila melanogaster*), and human (*Homo sapiens*), and built an interactome based on orthologs predicted using InParanoid (Ostlund et al., 2010). This interactome is now in version 2.0 and distributed with the latest TAIR 10 release (Lamesch et al., 2012). Software tools and web servers have now been made available to enable researchers to implement the “interologs” strategy, for example see Gallone et al. (2011). The second strategy does not rely on any other species, but solely on genomic/proteomic/transcriptomic features of *A. thaliana* (Cui et al., 2008; Brandao et al., 2009; Lin et al., 2010; Gu et al., 2011). For example, in the work by Lin et al. (2009), 14 features including gene expression and domain interactions were extracted to construct positive/negative training sets, and support vector machines were built to recognize the “pattern” of interaction. Normally this type of strategy is more computationally demanding, as it needs to employ machine learning techniques in an iterative manner.

Following the production of an interactome for the model plant *A. thaliana*, the next challenge is to develop similar interactomes for crop plants. The close relationship between *Brassica* crop species and *A. thaliana* (Lagercrantz et al., 1996; Trick et al., 2009; Wang et al., 2011) provides an opportunity to infer the *Brassica rapa* interactome by utilizing the substantial amount of PPI data available for *A. thaliana*. Despite large amounts of experimental and predicted PPI data for *A. thaliana*, as of June 2012, no interactions were recorded in the NCBI Entrez gene DB for *Brassica* sub-species (Taxid 3705). Here, we have constructed the inferred *B. rapa* interactome based on *A. thaliana* PPI data from two resources: (i) *A. thaliana* PPI data from three major DBs, BioGRID, IntAct and TAIR; and (ii) ortholog-based *A. thaliana* PPI prediction data (Geisler-Lee et al., 2007). Linking between *B. rapa* and *A. thaliana* was accomplished in three ways: (i) ortholog prediction using InParanoid, (ii) identification of gene duplications in the Plant Genome Duplication Database (PGDD; Tang et al., 2008), and (iii) BLAST sequence similarity search. In addition, we followed a complementary approach, by looking at the specificity of PPI data at the level of domains. Domains are evolutionarily conserved protein subunits and earlier studies have shown that their interactions are also conserved across species, in a manner that is more conserved than the PPIs themselves, and that these domain pairs can be used as building blocks of the PPIs (Itzhaki et al., 2006; Schuster-Bockler and Bateman, 2007). Here we used the repertoire of domain–domain interactions (DDIs) inferred from *A. thaliana* PPI data, using the message-passing (MP) algorithm (Iqbal et al., 2008) to predict novel protein interactions in *B. rapa*, as well as to validate and examine the specificity of PPIs predicted using other orthology-based methods mentioned above. We also compared and combined these DDI data with experimentally observed and computationally predicted interacting domain data from the Database of Protein Domain Interactions (DOMINE; Yellaboina et al., 2011). Briefly, Pfam domains were assigned to each *B. rapa* protein using the HMMER software (Finn et al., 2010, 2011). By combining the MP algorithm with extant information based on DOMINE, we were able to predict PPIs from protein domain information.

In constructing the interactome, special attention was paid to the fact that *B. rapa* and *A. thaliana* genes/proteins do not necessarily follow a simple one-to-one relationship. Although sequencing of the *B. rapa* genome has confirmed its almost complete triplication relative to *A. thaliana*, since formation of the postulated original hexaploid ancestor, substantial gene loss (fractionation) has occurred, and *B. rapa* contains 41,174 identified protein-coding genes compared with 33,602 in *A. thaliana* (Wang et al., 2011; Lamesch et al., 2012). In addition, it is worth noting that of a total of approximately 17,000 *B. rapa* gene families, only 5.9% appeared to be lineage-specific, with 93% shared with *A. thaliana* (Wang et al., 2011). When considering the possibility of functional divergence of genes which are duplicated/triplicated in *B. rapa* relative to *A. thaliana*, it is also worth noting that duplicated genes encoding products which interact with other proteins or are part of networks may be expected to be less likely to diverge than those which are less well connected (Zhang et al., 2005).

The inferred *B. rapa* interactome presented here, together with the *B. rapa* (Chiifu-401-42) genome sequence (Wang et al., 2011), provide a useful starting point for functional PPI studies and knowledge transfer from the model plant *A. thaliana* to *Brassica* crop species. One such example is the EU PP7 project MEIOsys (Systematic Analysis of Factors Controlling Meiotic Recombination in Higher Plants), which is aimed at identifying factors controlling crossover frequency and distribution in higher plants. This project uses affinity-based techniques to isolate meiotic protein complexes from *Brassica oleracea* for analysis by mass spectrometry (Osman et al., in press). For this, the *B. rapa* (Chiifu-401-42) genome sequence and the predicted interactome presented in this paper have already proved to be valuable resources, facilitating the screening of *B. oleracea* peptides for protein identification and the identification of possible PPIs. As such, we believe that the predicted interactome is also a useful resource for the wider *Brassica* research and crop-breeding community.

## Materials and Methods

### Accessing PPI DBs

Usually PPI DBs provide a web-interface, where an individual or list of protein/gene IDs can be used to query the DB. Some DBs can also be downloaded in a customized format for further investigation, e.g., the Database of Interacting Proteins (Xenarios et al., 2002). An increasing number of DBs also provide a version that complies with the Proteomics Standards Initiative – Molecular Interaction (PSI-MI) standard format (Kerrien et al., 2007). However, implementations of the PSI-MI format differ slightly from each other, which limit the reusability of existing codes. As a recent effort, PSI common query interface (PSICQUIC) was introduced (Aranda et al., 2011), which aims at providing a uniform query access for different PPI DBs. Queries to supporting DBs can be performed over the web in a manner as if it was a single DB. However, querying and compiling these DBs remains a challenging task, especially for large data sets, because, for example, different DBs use different unique IDs.

**Figure 1:**
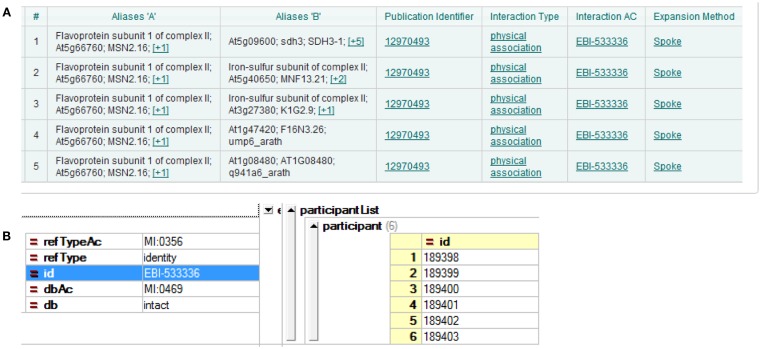
**Different interpretations of experimental data result in different DB formation**. **(A)** Using the spoke model, interaction EBI-533336 shows as five binary interactions on the IntAct website; **(B)** the same interaction shows in PSI-MI XML format as an interaction with six participants.

Three major *A. thaliana* PPI DBs were used in the current study: BioGRID, IntAct, and TAIR. The most recent versions at the time of the analysis were BioGRID 3.1.87, IntAct 2012-03-15, and TAIR 10. The DBs were presented according to different interpretations of experimental results. The simplest case is yeast two-hybrid, where two proteins form a direct binary/pairwise interaction. Other methods of analysis, for example co-immunoprecipitation, can identify protein complexes, which result in more complicated forms of representation of the DB. A popular choice of representation is the spoke model, in which such experimental results are interpreted as a set of binary interactions between the bait protein and co-precipitating proteins. Another form of representation, so called “matrix form,” assumes all co-precipitating proteins form binary interactions with each other. But this representation is considered less accurate (Bader and Hogue, 2003; Lysenko et al., 2009). Examples of both can be seen in Figure 1. In the current study, we downloaded all DBs in the PSI-MI TAB format, which uses the spoke model (Kerrien et al., 2007).

### PPI data compilation

**Figure 2:**
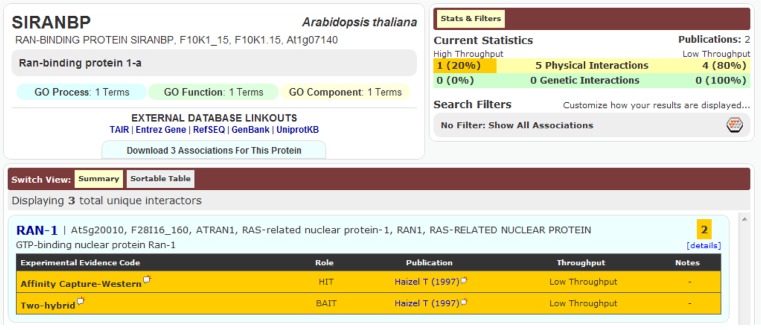
**Interaction between SIRANBP (At1g07140) and RAN-1 (At5g20010) was recorded in BioGRID as two separate entries because they were detected using two different methods, despite being from the same publication**.

An important aspect of a PPI is its detection method. Accordingly, if the same binary interaction was detected using different methods, or in different studies, all three DBs mentioned would list these binary interactions as separate entries. An example of this is seen in Figure 2. Although the detection method provides extra information for the DBs, in the current circumstances it leads to duplication and was thus removed during our data preparation. In fact, during the pre-processing of these DBs, we kept only the information of the two partners involved in the binary interaction, along with the original publication where the experiments appeared (i.e., PMID number); all other information provided with the PSI-MI TAB format was removed.

**Figure 3:**
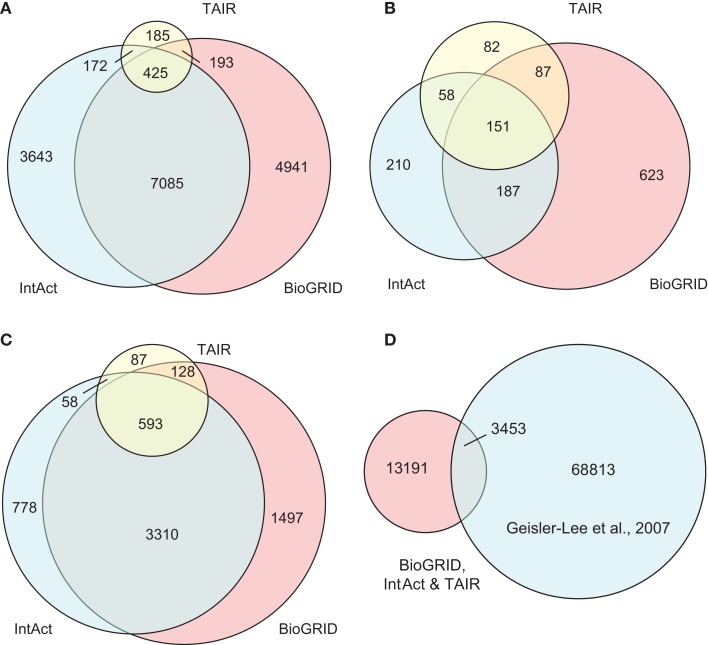
**Contributions of the three source DBs to the compiled data set (D1) in terms of: (A) binary interactions, (B) publication, and (C) interacting proteins**. The largest contribution comes from BioGrid, although the overlap among the three is significant. **(D)** A small part of interaction predictions made by Geisler-Lee et al. (2007) were confirmed in PPI DBs, while the remaining form the complementary source of PPI data in our study. Venn diagrams showing correct proportions were drawn using Venn Diagram Plotter Pacific Northwest National Laboratory, http://omics.pnl.gov/software/VennDiagramPlotter.php

The compiled *A. thaliana* PPI data (denoted by D1) consists of 16,644 binary interactions from 1,398 published research articles. The total number of proteins involved in D1 is 6,451, which does not include splicing variants. The contributions of the three source DBs to D1 can be seen in Figure 3. BioGrid is the largest source of interactions, followed by IntAct. Although TAIR is the smallest DB, it contains records complementary to the two main resources, and so is still valuable. It is interesting to note that although there were significant overlaps among the three DBs in terms of binary interactions and interacting proteins (Figures 3A,B), it seems that the overlap in terms of publication is not significant (Figure 3C). This highlights the importance of multiple data sources in the PPI prediction.

Besides experimentally verified PPIs from the three DBs, predicted PPIs were also used in our study. Geisler-Lee et al. (2007) studied PPIs in four model organisms, and predicted 72,266 PPIs based on interologs. Thus far, with information from recent publications, 3,453 of these have been confirmed. For example, the predicted interaction between AtSPO11-1 (At3g13170) and AtPRD1 (At4g14180) was later confirmed by yeast two-hybrid assay (De Muyt et al., 2007) and recorded under ID EBI-1540718 in IntAct. The remaining 68,813 PPIs that are yet to be confirmed were used in the current study as a complementary PPI source, denoted by D2. The relationship between the data compiled from the three DBs (i.e., D1) and the prediction made by Geisler-Lee et al. (2007) can be seen in Figure 3D.

### Linking the two species

The objective of the present research is to use known *A. thaliana* PPI data in order to expand the predicted *B. rapa* interactome. It is vital that the links between the two species are established correctly. An obvious way of achieving this is to identify orthologs between them. Using InParanoid, a total of 17,859 orthologous clusters were detected, which contain 18,830 and 21,873 proteins for *A. thaliana* and *B. rapa* respectively. Note that the number of orthologous clusters is less than the number of proteins for both species. This is a desirable feature as it may be indicative of possible gene duplication events within each species. Thus, in terms of DB implementation, this creates multi-to-multi relationships within the orthologous clusters.

In general, ortholog prediction methods can be classified into two broad categories: methods based on pairwise alignments, for example InParanoid, and methods based on phylogenetic trees (Kuzniar et al., 2008). The pairwise alignment methods have been found to outperform tree-based methods (Ostlund et al., 2010), which is why they were adopted in the current study. A complementary way of identifying related proteins, however, is to look at synteny and collinearity. In fact, since the release of the *B. rapa* genome sequence, several comparative genomics DBs (Lyons and Freeling, 2008; Tang et al., 2008; Tang and Lyons, 2012) have made use of the sequence. One of these, PGDD (Tang et al., 2008), identified 682 gene/protein blocks between *A. thaliana* and *B. rapa*, each of which consists of the same number of genes/protein from both species. PGDD allows a single gene/protein to appear in several different blocks. This effectively creates a multi-to-multi relationship. The total number of proteins covered in PGDD is 18,207 and 27,536 for *A. thaliana* and *B. rapa* respectively. Combining InParanoid and PGDD, a “bridging” DB was obtained, covering 21,624 and 31,423 proteins for *A. thaliana* and *B. rapa* respectively.

The total number of protein-coding genes released in the *B. rapa* sequencing project is 41,173. This leaves 9,750 *B. rapa* proteins that are not associated with any partners in *A. thaliana*. Therefore, we performed a BLAST similarity search using these 9,750 proteins against *A. thaliana* with a cut-off *e*-value of 1.0e−6. It was found that 7,307 had a hit in *A. thaliana* and interestingly, 1,376 hits reported an *e*-value of 0 (i.e., too small to report). These one-to-one data were then added to the previously compiled set to form the final “bridging” DB, denoted as D3. *B. rapa* proteins not covered by D3 account for approximately 5.93% (2,443/41,173). This is in agreement with a previous study which found that 95.8% of gene models have a match in at least one of the public protein DBs (Wang et al., 2011).

### *B. rapa* protein domain assignments

The total number of *B. rapa* proteins covered by D3 was 38,730, which still falls short of the *B. rapa* total of 41,173. To predict possible interactions for those *B. rapa* proteins that do not have counterparts in *A. thaliana*, as well as to complement the above mentioned methods of interactome prediction, we used other means of prediction in building the final interactome, i.e., looking at the level of DDIs. This not only increases the coverage of the interactome, but also gives a higher level of confidence. In addition, it provides more detailed information concerning which domains are potentially mediating the protein interactions. For this purpose, *B. rapa* protein domain assignments and interacting domain data (inferred using PPI data from *A. thaliana* as well as known domain interactions) can be used to predict possible protein interactions. HMMER (Finn et al., 2011) was used to search *B. rapa* protein sequences against the Pfam-A DB (Finn et al., 2010), using stringent criteria (*e*-value = 1.0e−10). As a result, 3,482 Pfam-A domains were assigned to 27,452 *B. rapa* proteins. On average, we had 1.43 domains assigned to each *B. rapa* protein. This is comparable with the TAIR Pfam annotation (1.41 domains/protein).

### DOMINE: The interacting domain database

The DOMINE DB (Yellaboina et al., 2011), which contains both experimental and predicted DDIs, was used in combination with the above mentioned *B. rapa* domain assignments. Here we used only known (i.e., observed) and high confidence predictions from DOMINE, which accounts for 8,173 unique interacting domain pairs. Known interacting domain data in DOMINE come from iPfam and 3did (Stein et al., 2011). With the release of Pfam version 26.0, additional entries were added. Fusing these entries together with DOMINE, we obtained 8,366 unique interacting domain pairs (denoted D4).

### The MP algorithm and training sets

Since *B. rapa* and *A. thaliana* are closely related, it is reasonable to assume that some interacting domains are conserved between the two species. In order to predict novel interacting domains, we employed the MP algorithm (Iqbal et al., 2008). MP is a popular method in the statistical inference community and has been applied in many hard inference problems in many fields (Berendsen et al., 1995; Richardson and Urbanke, 2001). Given the set of interacting and non-interacting protein pairs and their domain assignments, the MP method models this data as a factor graph which has two types of nodes: variable nodes which are the domain–domain pairs, and function nodes which are protein pairs (either interacting or non-interacting). The function nodes put constraints on the underlying variable nodes, as follows:

- For an interacting protein pair, at least one of the underlying domain pairs must be interacting.
- For a non-interacting protein pair, none of its underlying domain pairs should be interacting.

Given the existence of false positives in PPI data and our hypothesized negative data, the above constraints need to be “softened” to take into account the errors in the interaction map. This error is incorporated via an additional parameter ε, which ranges between 0 and 1 and quantifies our confidence in the PPI data (ε = 0 means the PPI network is 100% reliable). Another parameter, the *a priori* probability (β*)*, takes into account any prior knowledge of the DDIs. Given the above constraints, the goal is to assign 1 s and 0 s to the domain pairs such that the maximum number of constraints is satisfied. For that purpose, under this factor graphical modeling framework, a powerful statistical inference method, belief propagation (BP), is employed to infer the domain–domain interaction probabilities.

Belief propagation performs exact inference if the underlying graph is a tree, which corresponds to the global minimum of a function, called Bethe free energy (Yedidia et al., 2005). Bethe free energy is a function of beliefs, which in our case are domain interaction probabilities. It has been shown that, even in the case of graphs with cycles, on convergence solutions obtained by BP correspond to the local minimum of Bethe free energy. Hence, as in Iqbal et al. (2008), an inference scheme using BP is used here by minimizing Bethe free energy which helps to estimate two known parameters in our model, i.e., ε and β. For details of the MP algorithm and BP, see Iqbal et al. (2008).

The input to the algorithm is an interaction map among a set of proteins, and a set of domain assignments for the relevant proteins. The output is a list of probabilities of interaction between each pair of domains. Domain assignments for *A. thaliana* were taken from the Pfam DB (Finn et al., 2010). The PPI data compiled previously were used as positive inputs. However, not all interaction detection methods accurately detect binary interactions, for example HTP (Lin et al., 2009). To minimize false positives and also to reduce the computational burden, only a subset of D1 (yeast two-hybrid data) was used (denoted D1-sub). The MP algorithm also requires negative samples, i.e., non-interacting protein pairs. It is difficult to build an accurate set of negative samples because it is inherently impossible to exclude non-interacting protein pairs with certainty, and hence such results do not usually appear in the literature. Researchers have used various methods for constructing “hypothetical” non-interacting protein pairs, for example those based on randomness or proteins separated in different subcellular localizations (Xu et al., 2010). In the current study, we adopt a random approach, with additional stricter rules. Two random proteins were taken to be non-interacting if: (i) they do not appear in D1, (ii) their domain pairs do not appear in D4, (iii) they must have the same GO term in terms of cellular component, and (iv) the absolute value of their co-expression is less than 0.4. The last two restrictions ensure that expression patterns of the two proteins/genes do not imply interaction (Allocco et al., 2004). The gene expression data were from ATTED-II (Obayashi and Kinoshita, 2010). As a result, 25,246 domain pairs and 9,076 positive/negative training samples were fed into the algorithm to make interaction domain predictions. The negative samples were denoted D5.

## Results and Discussion

**Figure 4:**
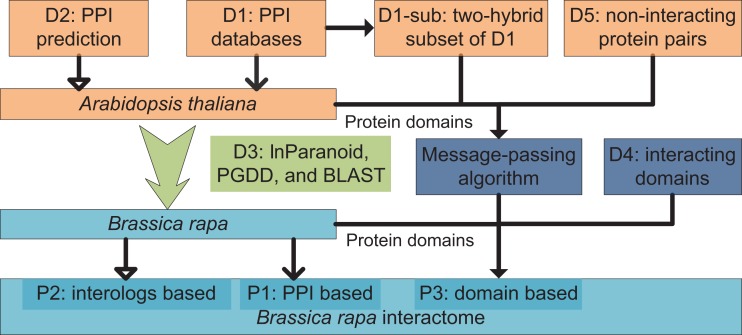
**Overview of data and methods used in the present study**. Three sets of interaction predictions were obtained: P1, PPI based; P2, interologs based; and P3, domain-based.

An overview diagram illustrating data and methods used in the present study is shown in Figure 4. Three sets of *B. rapa* interaction predictions were obtained: PPI based interaction (denoted P1), interologs based interaction (P2), and interacting domain-based interaction (P3). P1 and P2 were obtained using physical and predicted PPI data in D1 and D2, and the “bridging” DB D3. P3 were obtained using *B. rapa* protein domain assignments and the interacting domain data, which combine both “generic” known/high confidence interacting domain data in D4, and the *A. thaliana* “specific” interacting domain predictions using the MP algorithm and D1-sub/D5.

Restriction rules were applied to P3 to reduce the number of predictions and also increase the reliability: (i) two proteins in the pair need to share the same Gene Ontology (GO) cellular component terms in order for the domain-based prediction to take effect; (ii) if not predicted to be interacting in P1 or P2, a protein pair needs to have more than one interacting domain pairs; (iii) if predicted to be interacting in P1 and P2, a protein pair can have only one interacting domain pair. GO terms were assigned to *B. rapa* sequences using Argot2 (Fontana et al., 2009) with a stringent “internal confidence” value of 0.55, based on sequence similarity (UniProtKB/Swiss-Prot) and protein domain information (Pfam-A).

### Novel interacting domains

**Figure 5:**
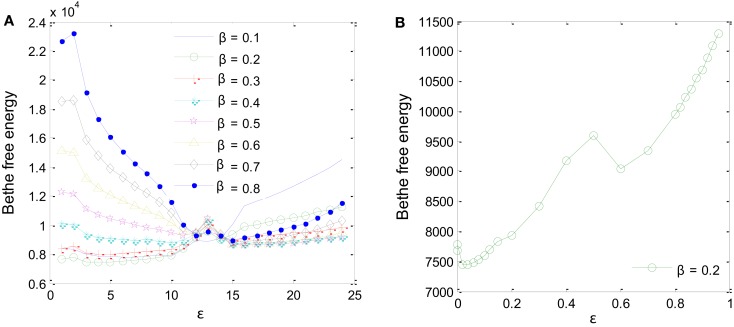
**Parameters for the MP algorithm had to be fine-tuned**. **(A)** *A priori* probability β = 0.2 produces the minimum Bethe free energy. **(B)** For β = 0.2, minimum Bethe free energy was reached at ε = 0.02.

Two parameters had to be fine-tuned for the MP algorithm to work correctly: the *a priori* probability, β, and the degree of reliability of the interaction datasets available for the inference, ε (Iqbal et al., 2008). Different values of β and ε were tested using training samples D1-sub and D5 to minimize Bethe free energy (Yedidia et al., 2005) as in Figure 5. For β values ranging from 0.1 to 0.8, a minimum Bethe free energy was reached for β = 0.2 (Figure 5A). Examining details of the minimum point, it was found that ε is equal to 0.02 (Figure 5B). These two values were taken forward to produce the final results.

The algorithm assigned probabilities of interactions to all 25,246 domain pairs. Special attention was paid to determine the cut-off value; on the one hand, a higher cut-off probability produces more reliable results but conversely it will produce fewer interacting domains, which does not fully represent the training sample. In the present study, a cut-off of 0.85 was used to select 2,389 high confidence interacting domain predictions. It was found that among these 2,389 domain pairs, 182 were also present in D4 (i.e., they were either physical interacting domain pairs observed in iPfam/3did, or high confidence predictions in DOMINE). A large proportion of these domain pairs (2,283) are the only domain pair in their respective protein pair in the positive training set D1-sub. They were successfully recognized; for example, domain pair PF01627 and PF03962 in protein pair AHP2 (At3g29350) and AtMND1 (At4g29170). (Interactions between AHP2 and AtMND1 were recorded under ID BIOGRID: 337481 and EBI-1555097). These predictions were considered unique contributions of the MP algorithm, and possibly conserved between *A. thaliana* and *B. rapa*. Combining results from the MP algorithm and D4, 10,573 unique interacting domain pairs were used to make prediction P3.

### The predicted interactome

**Figure 6:**
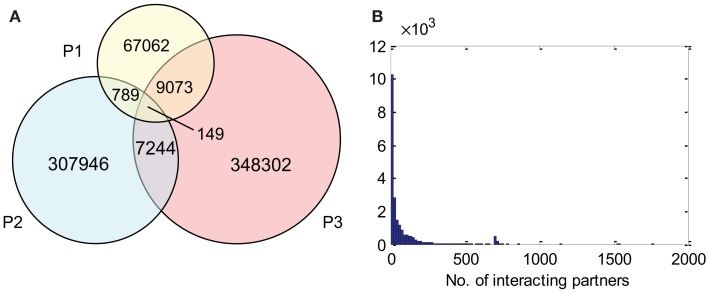
**(A)** The relationship between different prediction subsets. **(B)** Histogram showing degree distribution (number of interacting partners for each protein) for P-all.

P1, P2, and P3 contain 77,073, 316,128, and 364,768 predicted interactions respectively; all three datasets gave a total number of 740,565 unique predicted interactions (the predicted *B. rapa* interactome, denoted by P-all). The relationship among the three sets is shown in Figure 6A. The histogram of the number of interacting partners for each protein in P-all is shown in Figure 6B. The peak in Figure 6B is the first bin (i.e., degree < 10), which contains nearly half of proteins present in P-all (10,254 vs. 20,677). It is also worth noting that there are a small number of protein “hubs” with interacting partners between 700 and 1,774. These hubs may be important because they link the network together. On average, each protein in P-all interacts with 71 partners, which is higher than the estimation that a single protein interacts with about 5–50 proteins (Deng et al., 2002). The group of the 10 most connected hubs of P-all are shown in Table 1, which based on their known functions is not unexpected. Furthermore, some in this group do not have symbols, indicating that they have not been experimentally identified.

**Table 1:** **Top 10 interaction hubs of P-all and their *A. thaliana* counter parts**.

*B. rapa*	Interactions	*A. thaliana*	Resources	Symbols	Description
Bra014387	1774	At2g47610	I		Ribosomal protein L7Ae/L30e/S12e/Gadd45 family protein
		At3g62870	P		Ribosomal protein L7Ae/L30e/S12e/Gadd45 family protein

Bra003119	1540	At5g52640	P	HSP81-1	Heat shock protein 90.1

		At1g65350	I	UBQ13	Ubiquitin 13
		At3g09790	I, P	UBQ8	Ubiquitin 8
		At4g02890	I	UBQ14	Ubiquitin family protein
Bra009542	1144	At4g05320	I	UBQ10	Polyubiquitin 10
		At5g03240	I, P	UBQ3	Polyubiquitin 3
		At5g20620	I	UBQ4	Ubiquitin 4
		At5g37640	I	UBQ9	Ubiquitin 9

Bra024839	867	At2g01950	B	VH1	BRI1-like 2

Bra024840	867	At2g01950	B	VH1	BRI1-like 2

Bra016839	794	At1g11320	P		Unknown protein

Bra032392	759	At1g30470	P		SIT4 phosphatase-associated family protein

Bra021474	755	At3g02200	P		Proteasome component (PCI) domain protein
		At5g15610	P		Proteasome component (PCI) domain protein

Bra013661	740	At4g22930	P	PYR4	Pyrimidin 4

Bra036269	738	At4g02410	B		Concanavalin A-like lectin protein kinase family protein

*B, BLAST; I, InParanoid; P, PGDD*.

The three sets of PPI predictions constitute two levels of confidence of the predicted interactome. The high confidence prediction (Phc) has support from at least two sources of evidence, the low confidence prediction (Plc) has support from only one. Phc and Plc contain 17,255 and 723,310 interactions respectively. Some structural properties depicting P-all and the two different confidence level sub-networks were calculated using R package igraph (Csardi and Nepusz, 2006), as seen in Table 2. In all three cases there were large numbers of self-interactions. While these self-interactions constitute an important aspect of the interactome, they were removed from further analysis of the network structure. Interestingly, the network diameter (largest distance between two proteins) and the averaged shortest path length for Phc were significantly larger than those of Plc. This suggests that Phc contains a large sparsely connected network. It was also interesting to note that the average number of interacting partners, transitivity (i.e., clustering coefficient) and centralization of Plc are dramatically larger than those of Phc. This indicates that although Plc may contain less confident predictions, it is still useful in that it gives a densely connected network that contains all possible interactions.

**Table 2:** **Structural properties depicting the interactome P-all and two confidence levels of the sub-network: high confidence (Phc) and low confidence (Plc)**.

	Phc	Plc	P-all
No. of proteins	4,483	20,537	20,677
No. of interactions	17,255	723,310	740,565
No. of isolated proteins (ignore self-interaction)	155	50	54
No. of self-interaction	1,881	5,367	7248
No. of protein clusters	628	116	129
Diameter	32	10	11
Averaged neighbors	6.86	69.92	70.93
Averaged shortest path length	10.64	3.62	3.61
Transitivity	0.58	0.75	0.75
Centralization	0.01	0.08	0.08
Density	1.72E−3	3.43E−3	3.46E−3

### Interactome coverage

**Figure 7:**
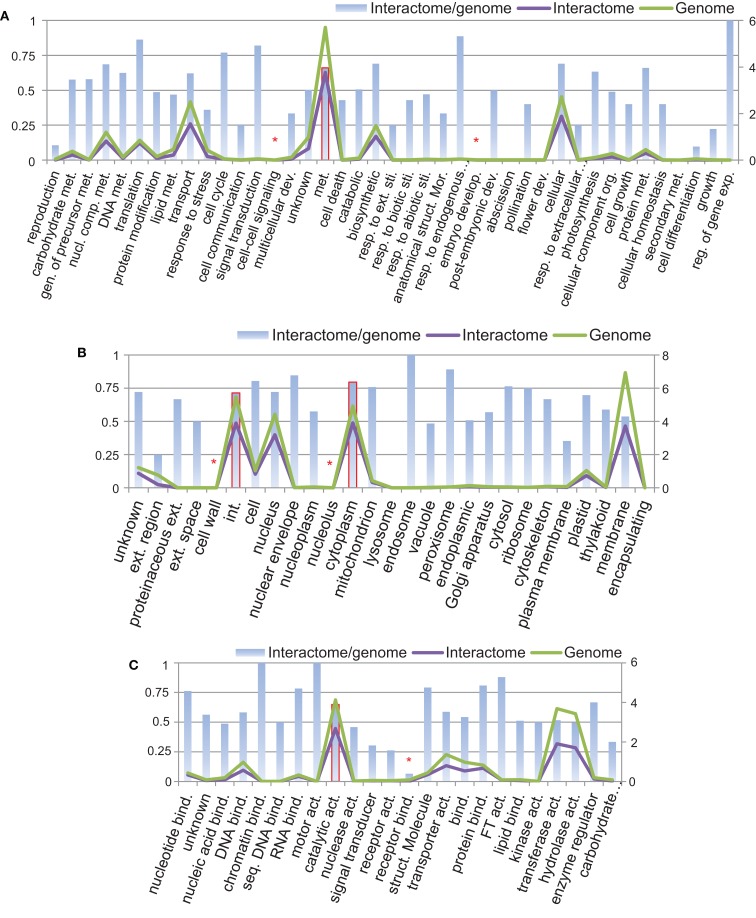
**Protein categories of the interactome in comparison with the whole *B. rapa* genome using GO plant slim categories: (A) biological process, (B) cellular component, and (C) molecular function**. Left vertical axis shows the interactome/genome percentage. Right vertical axis shows protein counts in units of 1,000. Abbreviations: act, Activity; bind, binding; comp, compound; dev, development; exp, expression; ext, extracellular; gen, generation; int, intracellular; met, metabolic; mor, morphogenesis; nucl, nucleobase; org, organization; reg, regulation; resp, response; seq, sequence-specific; sti, stimulus; struct, structure.

Using Argot2 (Fontana et al., 2009), 66% of all *B. rapa* protein-coding sequences (27,179/41,173) were assigned at least one GO term. We then categorized these proteins (i.e., genome) and the proteins from P-all (i.e., interactome) in terms of GO plant slim categories using AgBase (McCarthy et al., 2006). The results are shown in Figure 7.

From Figure 7 it is evident that in every category the number of proteins present in the interactome (purple line) follows the number of proteins in the genome (green line), and that in most categories the interactome/genome ratio is greater than 50% (bars). There are several categories with very small interactome/genome ratios, for example, cell–cell signaling and embryo development in the biological process category (highlighted by asterisk in Figure 7A), cell wall and nucleolus in the cellular component category (Figure 7B), and receptor binding in the molecular function category (Figure 7C). In these categories proteins do not count for a large number in either the genome or the interactome. On the other hand, most proteins from the interactome or genome fall into several specific GO slim categories, and have relatively high interactome/genome ratios. Those categories include metabolic process in biological process (highlighted by bars with solid borders in Figure 7A), intracellular and cytoplasm in the cellular component (Figure 7B), and catalytic activity in the molecular function (Figure 7C). From the above analysis, we concluded that the interactome is generally representative of the *B. rapa* genome. Given that a total number of 20,677 proteins are present in P-all, the protein coverage of the interactome is about 50%.

It is difficult to estimate the interaction coverage of the interactome. However, assuming the same rate of interaction as in *A. thaliana* (Lin et al., 2009), we estimated that there would be approximately 220,000 interactions for approximately 21,000 proteins in P-all. Thus the predicted interactome, with more than 740,000 interactions, is likely to have a very high false positive rate. On the other hand, the high confidence Phc contains 17,255 unique interactions, which would be coverage of approximately 78%, and thus is likely to be missing many true interactions. It is rare that, in terms of predicted interactomes, predictions match expectations exactly. For example, in PAIR (the predicted *Arabidopsis* interactome resource; Lin et al., 2009, 2010), the high confidence predictions are expected to cover 29.02% of the entire interactome. However, in the present study of *B. rapa* the problem of coverage/false positive rates seems to be exaggerated. The reasons for this are twofold: (i) Because of gene duplication/loss, genes of *A. thaliana* and *B. rapa* form a multi-to-multi relationship. However, in the interologs based prediction (P1 and P2), it is barely possible to rule out any predicted interactions (Pennisi, 2012). (ii) In the domain-based prediction P3, protein domains and GO terms were derived through computational predictions. However, parameters of the prediction algorithms, e.g., InParanoid/HMMER need to be fine-tuned to achieve higher accuracy. In addition, we used all physical interacting domain data from DOMINE, but it is possible that certain domains may only be interacting under certain cellular conditions. To address coverage/false positive rates issues, experiments need to be carried out to test predicted interactions in order that rules may be established to exclude any false positive predictions.

### Gene duplication and the “bridging” DB

The source data of the predicted *B. rapa* interactome came from *A. thaliana*. Thus it is vital that the relationships between the two genomes were correctly defined. Importantly, consideration must be given to the fact that there has been almost complete triplication of the *B. rapa* genome relative to *A. thaliana*, although since formation of the postulated original hexaploid ancestor, substantial gene loss has occurred (Wang et al., 2011). In this and the following sections we use known *A. thaliana* meiotic genes as an example to discuss gene duplication and its effect on the *B. rapa* meiosis network.

Meiosis is a key biological process that underpins sexual reproduction. During meiosis, a single round of DNA replication is followed by two rounds of nuclear division to produce four haploid gametes. Many genes/proteins participate in meiosis, for example, see reviews (Ma, 2006; Hultén, 2010; Osman et al., 2011). Here we used the list of 71 meiotic genes presented in (Yang et al., 2010), with the addition of *AtASY3* (At2g46980), recently described by the Birmingham meiosis group (Ferdous et al., 2012).

**Figure 8:**
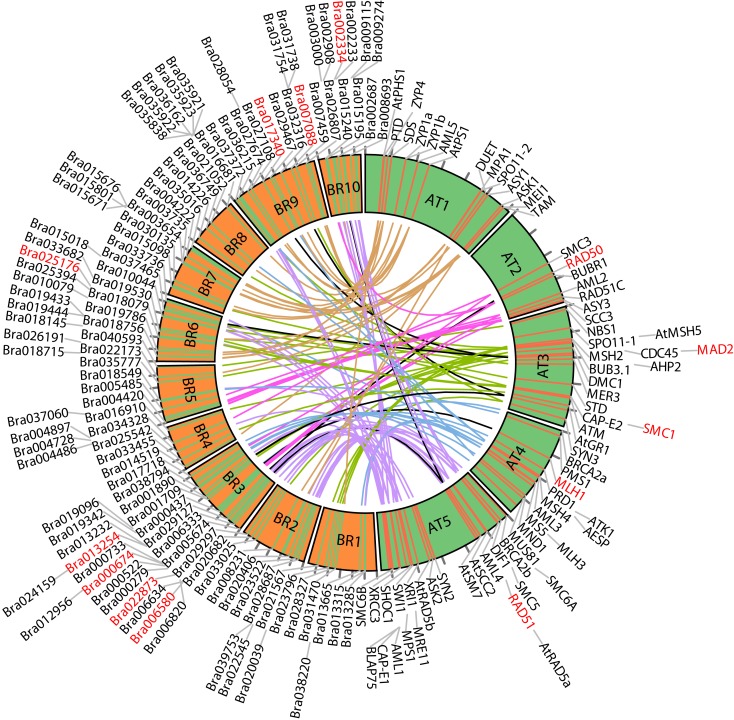
**Chromosome positions of 72 selected *A. thaliana* meiotic genes and their counterparts in *B. rapa***. The “bridging” DB represents possible collinearity and gene duplications. Genomes are arranged clockwise: *A. thaliana* chromosomes 1-5 (AT1 – AT5); *B. rapa* chromosomes 1-10 (BR1-BR10). Figure generated using Circos (Krzywinski et al., 2009).

For ease of interpretation we have presented the relationships between the two species in a one-to-multi manner from the *A. thaliana* perspective, as shown in Figure 8 and Table 3. Figure 8 shows chromosome positions of 72 known *A. thaliana* meiotic genes and their “counterparts” in *B. rapa*. It is evident that in our “bridging” DB there are conserved collinear blocks between the two genomes, for example, between the end of *A. thaliana* chromosome 2 (AT2) and the start of *B. rapa* chromosome 5 (BR5). This is in agreement with observations by Wang et al. (2011). Furthermore, we modeled possible gene duplications of *A. thaliana* meiotic genes, for example those on AT5 migrating to BR2/BR3/BR6/BR10.

**Table 3:** **Some known *A. thaliana* meiotic genes and their counterparts in *B. rapa***.

Protein locus	Gene name/chromosome	Pfam domain	GO: biological process	GO: cellular component	GO: molecular function	Resources
At2g31970	*RAD50*	Rad50_zn_hook, AAA_23, SbcCD_C	Telomere maintenance; DNA repair; double-strand break repair; mitotic recombination; telomere capping	Nucleus; cytoplasm; Mre11 complex	Nuclease activity; ATP binding; zinc ion binding	
Bra022873	BR3			nucleus		I, P

At3g25980	*MAD2*	HORMA	Mitotic cell cycle spindle assembly checkpoint	Kinetochore; chromocenter	DNA binding; protein binding	
Bra017340	BR9	HORMA	Cell cycle		Protein binding	I, P
Bra025176	BR6		Cell cycle		Protein binding	P

At3g54670	*SMC1*	SMC_N, SMC_hinge	Chromosome segregation; sister chromatid cohesion; chromosome organization	Nucleus; chromosome; cohesin complex; chloroplast	Transporter activity; protein binding; ATP binding	
Bra007088	BR9	SMC_N, SMC_hinge, AAA_23		Chromosome		I, P
Bra013254	BR3	SMC_N, SMC_hinge, AAA_23		Chromosome		B

At4g09140	*MLH1*	DNA_mis_repair, HATPase_c	ATP catabolic process; mismatch repair; mitotic recombination; reciprocal meiotic recombination; pollen development; seed germination; fruit development; seed development	Nuclear chromatin; synaptonemal complex; nucleus; chiasma; MutLalpha complex; MutLbeta complex	ATP binding; ATPase activity; protein binding, bridging; mismatched DNA binding	
Bra000674	BR3	DNA_mis_repair, HATPase_c_3		Nucleus		I, P

At5g20850	*RAD51*	Rad51	DNA metabolic process; DNA repair; double-strand break repair; regulation of transcription, DNA-dependent; response to radiation; response to gamma radiation	Nucleus	Nucleotide binding; DNA binding; damaged DNA binding; protein binding; ATP binding; DNA-dependent ATPase activity; nucleoside-triphosphatase activity	
Bra002334	BR10	Rad51, AAA_25	DNA metabolic process	Nucleus		P
Bra006580	BR3	Rad51, AAA_25	DNA metabolic process	Nucleus		I, P

*B, BLAST; I, InParanoid; P, PGDD*.

*Each group (separated by solid line) starts with an *A. thaliana* gene, followed by one or more *B. rapa* genes*.

Table 3 gives some detailed information for several meiotic genes presented in Figure 8, where related genes from the two species are grouped together. Each group is led by an *A. thaliana* meiotic gene, followed by its *B. rapa* counterpart(s) and the inference resources. We also listed domain (Pfam) and GO term names for these genes/proteins where available. We can see that quite often the relationships were confirmed by more than one method/resource. Furthermore, most related proteins have a similar domain structure, for example AtMAD2 and its counterparts in *B. rapa* (highlighted in Figure 8). However, in groups containing *AtSMC1* and *AtRAD51*, it seems that *B. rapa* genes have additional functions compared to their counterparts in *A. thaliana* (i.e., additional AAA_23 and AAA_25 domains respectively). For GO terms, as we used stringent criteria, fewer GO terms were assigned to *B. rapa* proteins. However, assigned terms mostly agree with their counterparts in *A. thaliana*.

### The meiosis network

**Figure 9:**
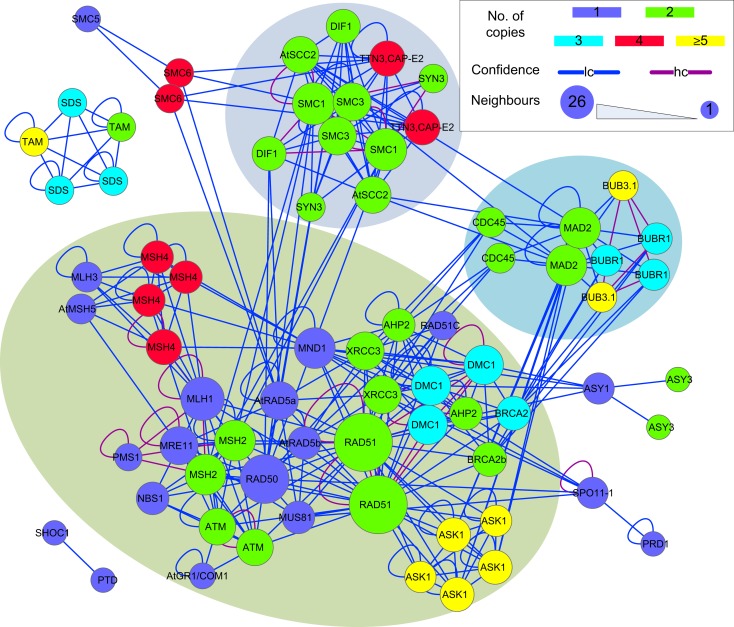
**A sub-network of the predicted interactome formed by putative *B. rapa* meiotic proteins**. It is a densely connected network containing several hub proteins. *B. rapa* proteins are shown by their *A. thaliana* counterparts’ names; their unique accession numbers are not shown. Node color denotes the number of copies that a single *A. thaliana* gene/protein has in *B. rapa*. The confidence of interaction prediction is presented by edge colors. Node size is set to be proportional to the number of interacting partners of each individual protein. Figure generated using Cytoscape (Smoot et al., 2011).

The sub-network formed by putative *B. rapa* meiotic proteins was extracted from P-all (Figure 9) as an example to demonstrate the utility of the predicted interactome. From Figure 9 it is obvious that there is a large number of putative *B. rapa* meiotic proteins which are sole copies of their *A. thaliana* counterparts. It is likely that these proteins are functionally identical to those in *A. thaliana*. Multi-copy proteins are also found and in some cases at least, their functions appear to have differentiated. For example, there are four *B. rapa* counterparts of AtSMC6, but two of them do not appear to participate in meiosis. However, for the majority of multi-copy proteins similar interacting partners are identified.

In terms of interactions, there were several hub proteins in the network, e.g., RAD51 *(26 connections*), RAD50 *(19 connections*), MLH1 *(15 connections*), SMC1 *(14 connections*), *and* MAD2 *(13 connections*). Interestingly, these hub proteins were identified by the MCL algorithm (Enright et al., 2002) to form separate clusters with their direct neighbors (shadowed areas in Figure 9). Most of the interactions in the network were supported by only one piece of evidence (low confidence), and high confidence interactions were sparse and mainly self-interactions. However, it is a more dense and complex network than those predicted for *A. thaliana* (Lin et al., 2009) and rice (Aya et al., 2011) meiotic proteins.

**Figure 10:**
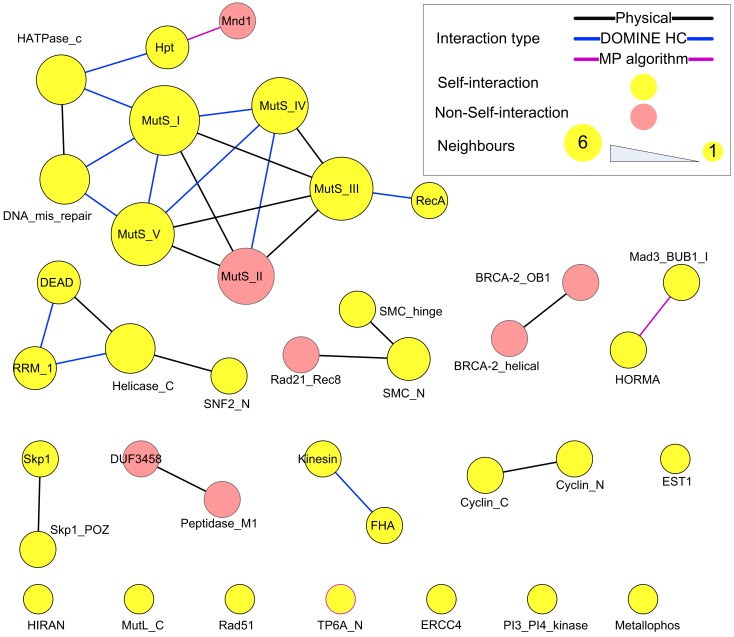
**Domain interactions contained in the putative meiotic network**. Types of “evidence” were shown as edge colors. Edges of self-interacting domains were omitted, but highlighted by node (and node border) color.

Protein domains contained in the putative meiotic network were extracted and their interactions are shown in Figure 10 (those of the hub proteins can be seen in Table 3). Overall, it is a sparsely connected network with mainly self-interactions. This suggests that although the meiotic protein interaction network has a very high density, the driving force mediating those interactions is possibly domain self-interactions. Most of the self-interactions are experimentally verified and some of them are derived from the MP algorithm, for example, self-interaction between TP6A_N. The biggest cluster was formed by the interactions among several domains, for example, MutS family domains (contained by MSH2, MSH4, MSH5), RecA (RAD51 and DMC1), and DNA mismatch repair (PMS1 and MLH1). Some of the proteins containing these domains are already thought to form protein complexes during meiosis. *In vitro* studies using purified human hMSH4 and hMSH5 have revealed that they act as complex to stabilize progenitor Holliday junctions (Holliday, 1964). Evidence suggests this is also likely the case in *A. thaliana*, for AtMSH4 and AtMSH5 (Higgins et al., 2004, 2008; Snowden et al., 2004). Other studies suggest that AtAHP2 (containing an Hpt domain) and AtMND1 (Mnd1) also form a complex (Vignard et al., 2007). During budding yeast (*Saccharomyces cerevisiae*) meiosis, interactions were found among MLH1, MLH3 (HATPase_c), and PMS1 (DNA mismatch repair and HATPase_c; Argueso et al., 2002; Nishant et al., 2008), however, these are yet to be experimentally verified in *A. thaliana*. Note that some of the self-interacting domains in Figure 10, for example TP6A_N (SPO11), do not show direct interactions with other domains. This does not necessarily mean that the interactome contains no predictions, but that for ease of visualization, we omitted indirect connections.

## Conclusion

In the present study, we have inferred the *B. rapa* interactome using PPI data available from *A. thaliana*. These PPI data were either physical interactions verified through experiments, or predictions based on orthology. The relationship between the two genomes was established by studying orthologs/collinearity/sequence similarity. We also utilized domain interactions in our predictions. Both known and predicted interacting domains, as well as protein domain assignments of *B. rapa*, were used to predict possible interactions.

The inferred interactome contains 17,255 predicted interactions at high confidence level, and 723,310 predicted interactions at low confidence level. The interactome covers around 50% of the proteins in the *B. rapa* genome, and its high confidence interaction predictions give a coverage of around 78% for those proteins. As a first effort of establishing a *B. rapa* interactome, our inferred interactome could be a useful resource for experimental biologists or other researchers using *B. rapa* as a working plant. The interactome is available at http://www.meiosys.org/dissemination/ as pure text files; other formats e.g., SQL are available upon request.

## Conflict of Interest Statement

The authors declare that the research was conducted in the absence of any commercial or financial relationships that could be construed as a potential conflict of interest.
